# The FGLamide-Allatostatins Influence Foraging Behavior in *Drosophila melanogaster*

**DOI:** 10.1371/journal.pone.0036059

**Published:** 2012-04-27

**Authors:** Christine Wang, Ian Chin-Sang, William G. Bendena

**Affiliations:** Department of Biology, Biosciences Complex, Queen's University, Kingston, Ontario, Canada; Ghent University, Belgium

## Abstract

Allatostatins (ASTs) are multifunctional neuropeptides that generally act in an inhibitory fashion. ASTs were identified as inhibitors of juvenile hormone biosynthesis. Juvenile hormone regulates insect metamorphosis, reproduction, food intake, growth, and development. *Drosophila melanogaster* RNAi lines of PheGlyLeu-amide-ASTs (FGLa/ASTs) and their cognate receptor, Dar-1, were used to characterize roles these neuropeptides and their respective receptor may play in behavior and physiology. Dar-1 and FGLa/AST RNAi lines showed a significant reduction in larval foraging in the presence of food. The larval foraging defect is not observed in the absence of food. These RNAi lines have decreased *for* transcript levels which encodes cGMP- dependent protein kinase. A reduction in the *for* transcript is known to be associated with a naturally occuring allelic variation that creates a sitter phenotype in contrast to the rover phenotype which is caused by a *for* allele associated with increased *for* activity. The sitting phenotype of FGLa/AST and Dar-1 RNAi lines is similar to the phenotype of a deletion mutant of an AST/galanin-like receptor (NPR-9) in *Caenorhabditis elegans*. Associated with the foraging defect in *C. elegans npr-9* mutants is accumulation of intestinal lipid. Lipid accumulation was not a phenotype associated with the FGLa/AST and Dar-1 RNAi lines.

## Introduction

In insects, three differing allatostatin (AST) peptide structures have been isolated that inhibit juvenile hormone (JH) biosynthesis. These include the FGLamide (FGLa)/ASTs, the W(X)_6_Wamide/ASTs and the PISCF/ASTs. Each unique peptide structure appears to inhibit JH biosynthesis in select insect species [1]–[3]. All three types of ASTs have been identified in *D. melanogaster*, but none have been identified as a regulator of JH biosynthesis [4]–[7].

The FGLa/ASTs are related to vertebrate somatostatin, galanin and opioid peptides and inhibit JH biosynthesis only in cockroaches, crickets, and termites [8]–[10]. In cockroaches, FGLa/ASTs also function to regulate gut contraction [11]–[13]. In *D.melanogaster* and other Diptera the FGLa/ASTs do not inhibit JH biosynthesis [7], [14]and their function has yet to be determined. *D. melanogaster* FGLa/ASTs functionally interact with two galanin-like receptors Dar-1 and Dar-2 [4], [15], [16]. Dar-1 is primarily expressed in the larval CNS whereas Dar-2 appears to be expressed in the crop, midgut and hindgut [17].

A *Caenorhabditis elegans* AST-like peptide receptor, NPR-9, was identified in a BLAST search as the closest related GPCR to Dar-1 [18]. Analysis of a deletion mutant of *npr-*9 revealed enhanced local search behavior and increased pivoting only in the presence of food. Mutant *npr-9* animals also displayed an increased level of intestinal fat. The foraging phenotype of *npr-9* is similar to a *D. melanogaster* mutation known as ‘sitter’ [19]. The sitter phenotype is due to a naturally occuring polymorphism in the *foraging (for)* gene. Two different alleles at the *for* locus characterize two different food-search behavioral phenotypes; *for* ^S^ *= *sitter, *for* ^R^ = rover. Rovers travel significantly greater distances when feeding as compared to the sitters, but no locomotor differences were seen between the two strains on non-nutrient media, suggesting that pathlength differences on food are not the result of a general locomotory defect [20]. The alleles differ by natural polymorphisms at the *dg2* (*for*) locus [21]. The *for* gene encodes a cGMP dependent protein kinase (PKG) and differences in PKG activity and *for* transcript levels are attributable to the differences in foraging behavior, where rovers have a significantly greater level of PKG activity and *for* transcript level when compared to sitters.

In this manuscript, we have identified an alteration in foraging behavior due to reduction in FGLa/ASTs or their receptor Dar-1. This is the first identified functional role for FGLa/ASTs or receptor Dar-1 in *D. melanogaster*.

## Materials and Methods

### Animals

*D. melanogaster* stocks were reared at 22°C, 12 hr light/dark cycle and 70%±5% relative humidity on standard medium containing 0.94% agar, 0.01% molasses, 8.2% cornmeal, 3.4% killed yeast, 0.18% benzoic acid, 0.66% proprionic acid. Gal 4-UAS RNAi transgenic lines were obtained from Vienna *Drosophila* RNAi Center (VDRC, Vienna, Austria) [22]. These lines were created by transformation of isogenic strain *w^1118^* which was used as a control in our experiments. Homozygous viable RNAi lines Dar-1 48496 and 101395 contained an insertion on chromosomes 1 and 2, respectively. Isogenic homozygous viable FGLa/AST RNAi lines 103215 and 14397 contained insertions on chromosomes 2 and 3, respectively. Dar-2 RNAi lines 1326 and 1327 were not used in this study as they showed slow developmental growth and feeding defects. The ubiquitous driver line daughterless (Da)Gal 4 and tissue specific driver 6986 were obtained from the Bloomington Stock center. The driver line 6986 expresses primarily in larval ring gland but also expresses in histoblasts, gut, Malpighian tubules, male accessory glands, testis sheath, and cyst cells [23].

### Larval Foraging Behavioral Assay

Third instar larval foraging assays from each cross (RNAi lines and *w^1118^* control crossed to DaGal4 or 6986 drivers) were examined using a modified procedure described [24], which is briefly outlined here. Third instar larvae (approximately 72 hours post-hatching) reared at 25°C were collected and washed with distilled water. Black rectangular Plexiglas plates (25 cm width×37 cm length×0.5 cm height) with 6 circular wells (0.5 mm deep with a 4.25 cm radius) were provided courtesy of the Sokolowski Lab (University of Toronto at Mississauga). Larvae were placed into the center of each of the 6 circular wells, which were filled with a thin layer of homogenized yeast paste (distilled water and Fleischmann's Bakers' Yeast; approximately 3∶1 ratio by weight). Wells were then covered with standard Petri dish lids. After 5 minutes the foraging path lengths made within the yeast were traced, scanned, and quantified using the ImageJ program (http://rsb.info.nih.gov/ij/).

Third instar larvae foraging behavior was also analyzed off food. Standard Petri dishes were used and filled with a thin layer of 2% agar containing neutral red dye. Larvae were placed into the center of one of these agar filled Petri dishes and the foraging path lengths traced after 5 minutes and analyzed the same way as the on food foraging assay.

### Triglyceride Extraction and Quantification

Triglyceride and proteins were extracted from third instar larvae as described [25]. Three independent extractions were used for quantification of triglyceride and protein. The triglyceride levels from the extracts were quantified using the TRIGs Kit (Randox). Protein levels were quantified using the BCA Protein Assay (ThermoScientific). Triglyceride levels were normalized to protein levels.

### RNA Extraction and Reverse Transcription

RNA was extracted from 30 mg of *D. melanogaster* third instar larvae from each RNAi, DaGal4 or 6898 driver control, and *w^1118^* lines using an RNeasy Kit (Qiagen). RNA was eluted with 30 µl of RNAse free water as opposed to the 50 µl suggested in the manual to concentrate the RNA extract further for reverse transcription. Remaining genomic DNA contamination was removed through the use of a DNA-free kit (Ambion), according to kit protocol.

All reverse transcription reactions were made with 8.0 µl volume of total isolated RNA at appropriate initial concentrations, as well as 83 µM dNTPs, 42 ng/µl oligo d(T), 3 µl of 5× RT Buffer, 40 U RNaseOUT (Invitrogen), 10.5 mM DTT, and 150 U of SuperScript II Reverse transcriptase (Invitrogen) into a final reaction volume of 15 µl. The following procedures and conditions were used for all reaction: first step was incubating RNA with a primer mix of oligo- d(T) and dNTPs at 65°C for 5 minutes, 3 minutes on ice and then a master mix of 5× RT Buffer, RNaseOUT, DTT was added, followed by an incubation step for 2 minutes at 42°C. SuperScript II reverse transcriptase was added to the mixture and a final incubation session for 50 minutes at 42°C was carried out. The reaction was then terminated by heat inactivation at 70°C for 15 minutes and chilled on ice for 3 minutes. Immediately after, 60 µl of autoclaved water was added to each reaction tube, resulting in a 5 fold-dilution of all cDNA samples.

### Quantitative PCR and Standard Curves using PCR Products

For all qPCR experiments, primers were set at a 700 nM concentration and added with 5 µl of cDNA and 1× volume of 2× qPCR MasterMix Plus for SYBR Green I Low ROX (Eurogentec) to bring the final volume to 25 µl for each reaction. A mastermix of SYBR green, primers, and water was prepared to minimize variation. All qPCR reactions were performed in an Applied Biosystems 7500 Real Time PCR System (Foster City, USA) under the following conditions: pre-PCR denaturation and polymerase activation step for 15 minutes at 95°C, followed by 45 cycles of 15 second denaturation at 95°C, 1 minute hybridization step at variable temperatures (see hybridization temperatures of select primers below), and a 36 second elongation step at 72°C. Dissociation curve analyses were done to confirm the amplification of a single PCR product, under the following real time conditions: 15 second denaturation step at 95°C, followed by 1 minute at 60°C, and 15 seconds at 95°C. The forward *for* primer 5′-ATTGTCGGGAGCGAAGGTC-3′ and the reverse primer 5′- ATGATGGTCTGAAAGCACTGG-3′ were used at a hybridization temperature of 62°C. The forward and reverse sequences for the Dar-1 primers were 5′-GCAGCCACTTATCGGTCATT-3′and 5′-CTTCCACACCAGACCACCTT-3′, respectively and the hybridization temperature used was 62°C. The forward FGLa/AST primer 5′- CTACGACCAGGACAACGAGA-3′ and the reverse primer 5′- CCCAGGCCAAAGTTGAAGG-3′ were used at a hybridization temperature of 62°C.The forward and reverse sequences for ribosomal protein gene *Rp49* were 5′-GACGCTTCAAGGGACAGTATCTG-3′ and 5′-AAACGCGGTTCTGCATGAG-3′, respectively and were used at a hybridization temperature of 56.8°C. Transcript levels for each gene were normalized to gene *Rp49* and presented as relative expression levels compared to a control except for *for* transcript levels which are presented as *for* expression levels normalized to *Rp49*.

### Statistical Analysis

For larval foraging behavioral assays, Image J was used to quantify foraging path lengths that were traced and scanned. T-tests assuming unequal variance were performed in Graphpad Prism to determine the statistical significance of the foraging path lengths, *for* transcript levels, and triglyceride levels between RNAi strains and their controls.

## Results

### Confirmation of RNAi Knockdown in Dar-1 and FGLa/AST RNAi

**Figure 1:**
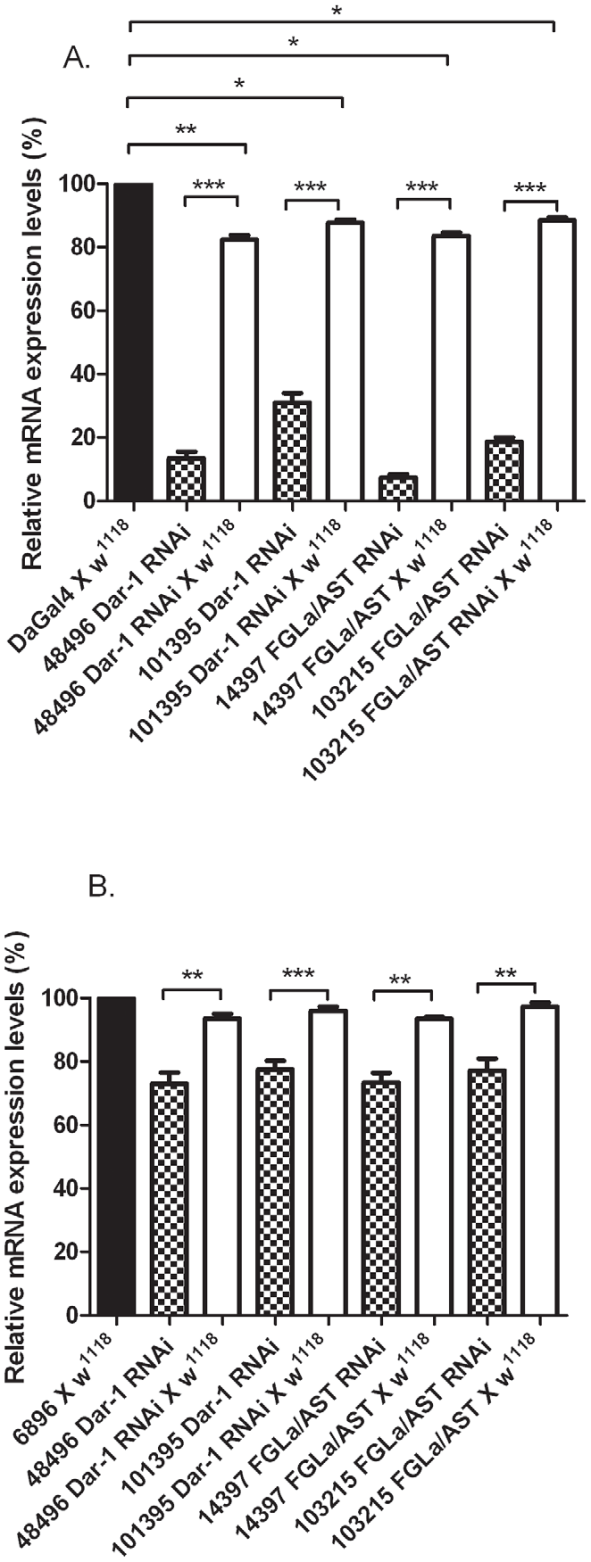
Relative mRNA expression levels of individual *D. melanogaster* stocks expressing RNAi to gene sequences for Dar-1 and FGLa/AST. **A**. Dar-1 or FGLa/AST mRNA levels were measured in DaGal4 x *w^1118^* and RNA levels in RNAi lines crossed to either DaGal4 (patterned bar) or *w^1118^*(white bar) were measured relative to this control. **B**. The same comparisons as in A. with driver line 6896 X *w^1118^* serving as the control. The number associated with each RNAi stock is the Transformation ID established by the Vienna *Drosophila* RNAi Center. Each bar represents two independent RNA extractions that were each assayed by qPCR three times. Thirty third instar larvae were used for each extraction. Expression levels were normalized using RP49 (ribosomal protein) as a standard. Asterisks indicate significant difference * = P<0.05; **P<0.001; ***P<0.0001.

To assess the level of mRNA reduction/knockdown in Dar-1 and FGLa/AST RNAi lines (VDRC transformant IDs: 48496 , 101395; and 103215, 14397 respectively), each were crossed to driver lines (DaGal4 or 6896) or to *w^1118^* . The relative transcript levels of each gene in the third instar larval stage was quantitated by real-time qPCR. Each Dar-1 and FGLa/AST RNAi line crossed to *w^1118^* (Figure 1A, white bars) was compared DaGal4 crossed to *w^1118^* (Figure 1A, black bar). In the absence of being crossed to DaGal4, the Dar-1 and FGLa/AST lines had significantly reduced RNA expression levels which ranged from approximately 12–18% (Figure 1A) suggesting that these RNAi lines without a driver exhibit some leaky gene knock down activity. When crossed to the DaGal4 each RNAi line exhibited a further significant suppression of mRNA levels. Relative to their respective RNAi lines crossed to *w^1118^*, DaGal 4 expression in 48496 and 101395 Dar-1 RNAi lines resulted in mRNA suppression by 70 and 56%, respectively. DaGal4 expression with FGLa/AST RNAi lines 14397 and 103215 resulted in mRNA levels being suppressed 76 and 70% respectively. Dar 1 and FGLa/AST RNAi were also crossed to a tissue selective driver line 6896 and each displayed a reduction in their respective target gene mRNA levels of approximately 20% relative to the same RNAi lines crossed to *w^1118^* (Figure 1B).

### Larval Foraging Assays forDaGal4 driven Dar-1 and FGLa/AST RNAi

**Figure 2:**
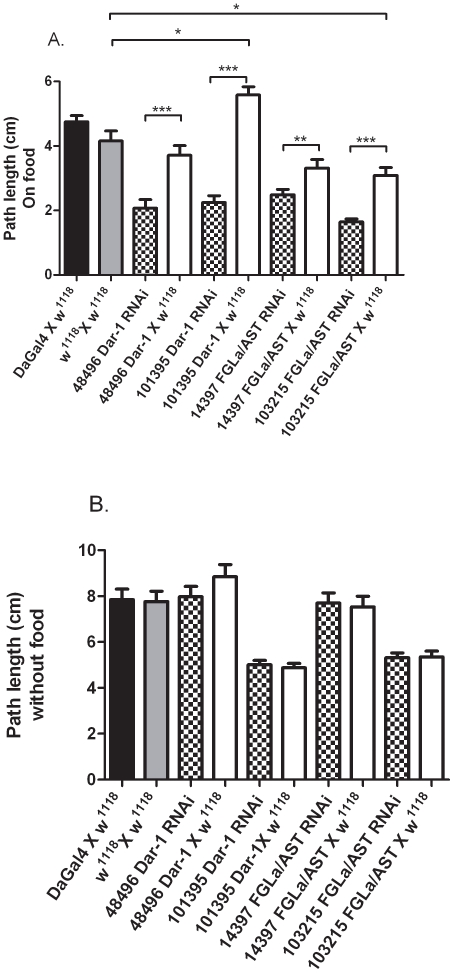
The foraging distance of third instar larvae (path length) A. in the presence of food and B. absence of food for 5 mins was measured for controls DaGal4 x *w^1118^*(black bar), *w^1118^* self-cross (grey bar) and Dar-1and FGLa/AST RNAi lines crossed to DaGal4 (patterned bar) or *w^1118^* (white bar); N = 30–34. Asterisks indicate a significant difference * = p<0.05;** = p<0.001 and *** = p<0.0001. Only the significance in comparison with *w^1118^* self-cross is indicated although comparison with DaGal4 X *w^1118^* was equivalent.

In the larval foraging assay, a self-cross of non-transformed *w^1118^* was compared to the driver DaGal4 X *w^1118^* to confirm that no statistical difference in foraging resulted from the introduction of the DaGal4 driver (Figure 2). A cross of each RNAi line with the DaGal4 driver (eg. 48496 Dar-1 RNAi) was then compared to a cross of each RNAi line with *w^1118^* in which RNAi should not be expressed (Figure 2A). On food, Dar-1 and FGLa/AST RNAi lines crossed to the DaGal4 driver showed significantly decreased foraging distances compared to the same RNAi lines crossed to *w^1118^*(Figure 2A). Similarly, on food foraging of Dar-1 and FGLa/AST RNAi lines crossed to the DaGal4 driver was significantly reduced relative to DaGal4 X *w^1118^* and the *w^1118^* self-cross (Figure 2A).

When tested in the absence of food, no significant difference in foraging behavior was found between self-crossed *w^1118^* and the DaGal4 driver line crossed to *w^1118^*. In the absence of food, no significant difference was found when RNAi lines for either Dar-1 or FGLa/AST crossed to the DaGal4 driver line were compared to their respective RNAi lines crossed to *w^1118^* (Figure 2B).

### Larval Foraging Assays for 6896 driven Dar-1 and FGLa/AST RNAi

**Figure 3:**
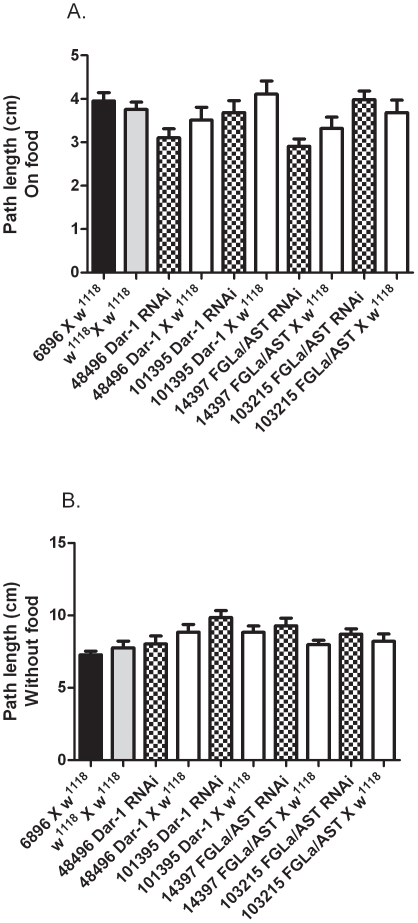
The foraging distance of third instar larvae (path length) A. in the presence of food and B. absence of food for 5 mins was measured for controls 6896 x *w^1118^*(black bar), *w^1118^* self-cross (grey bar) and Dar-1and FGLa/AST RNAi lines crossed to 6896 (patterned bar) or *w^1118^* (white bar); N = 30–34.

The foraging assay was repeated using third instar larvae from Dar-1 and FGLa/AST RNAi lines crossed to the tissue selective driver 6896. No significant reduction in foraging path length in the presence (Figure 3A) or absence (Figure 3B) of food was noted when 6896 X Dar 1 or FGLa/AST RNAi lines were compared to their respective RNAi lines crossed to *w^1118^*

### *for* Transcript Levels of Dar-1 and FGLa/AST Third Instar Larvae RNAi

Sitter and rover phenotypes differ in PKG activity and *for* transcript level, where sitters have lower PKG activity and *for* transcript levels compared to their rover counterparts [21]. This suggested that foraging defects in Dar-1 and FGLa/AST RNAi lines may result from reduced PKG due to alterations in the *for* transcript level. To examine this, we measured *for* transcript levels in each RNAi line.

**Figure 4:**
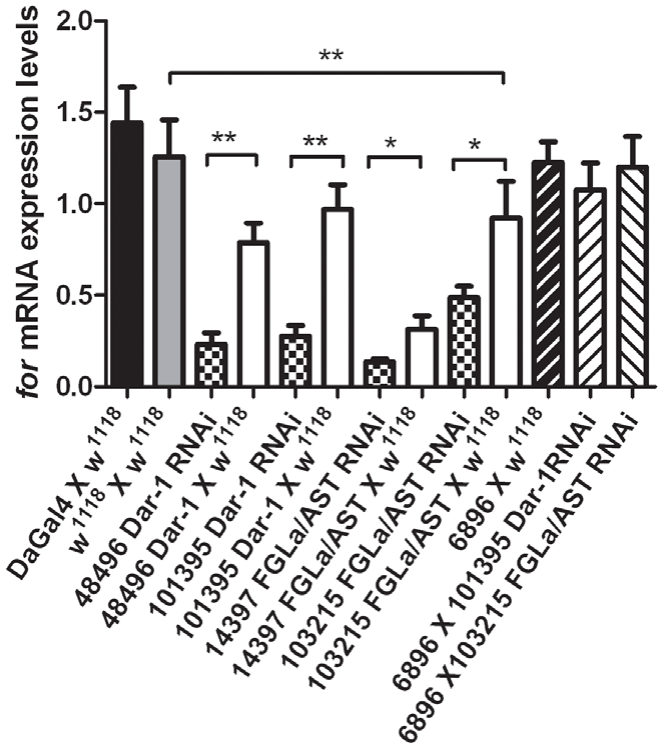
*for* mRNA expression levels of controls DaGal4 x *w^1118^* (black bar), *w^1118^* self-cross (grey bar), 6896 X *w^1118^* (black bar with white diagonal stripes) and experimental Dar-1 and FGLa/AST RNAi lines x DaGal4 (patterned bars) or *w^1118^*(white bars) and Dar1 and FGLa/AST RNAi lines x 6896 (white bars with black diagonal stripes). Each bar represents three independent RNA extractions that were each assayed by qPCR three times. Thirty third instar larvae were used for each extraction. Expression levels were normalized using RP49 as a standard. Asterisks indicate a significant difference * = p<0.05 and ** = p<0.001. Only the significance in comparison with *w^1118^* self-cross is indicated although comparison with DaGal4 X *w^1118^* was equivalent.

The *for* transcript levels of Dar-1 and FGLa/AST RNAi lines crossed to DaGal4 driver line were significantly reduced compared to the same RNAi lines crossed to *w^1118^* (Figure 4). RNA extracted from larvae of Dar-1 and FGLa/AST RNAi lines crossed to *w^1118^* have reduced *for* mRNA levels, with a significant reduction in the 14397 FGLa/AST RNAi line relative to DaGal4 X *w^1118^* and self-crossed *w^1118^* controls (Figure 4) which is consistent the RNAi lines without the drivers showing some knock down of the the Dar-1 and FGLa/AST genes. The tissue selective driver 6986 when crossed to *w^1118^*, 101395 Dar-1 RNAi, or 103215 FGLa/AST RNAi lines did not have any significant alteration in *for* mRNA levels relative to *w^1118^*self-cross (Figure 4). This is consistent with 6986 crosses failing to influence foraging behavior (Figure 3).

### Triglyceride Levels of Dar-1 and FGLa/AST Third Instar Larvae RNAi

*C. elegans npr-9* mutants showed both local search behavior defects and an increase in intestinal lipid accumulation compared to wild type worms [18]. Dar-1 and FGLa/AST RNAi lines showed foraging defects similar to that of *npr-9* mutants, which would suggest that foraging defects may be tied into decreased metabolic rate or increased food uptake efficiency. In order to assess this we measured the levels of total triglyceride in third instar larvae RNAi lines that showed foraging defects.

**Figure 5:**
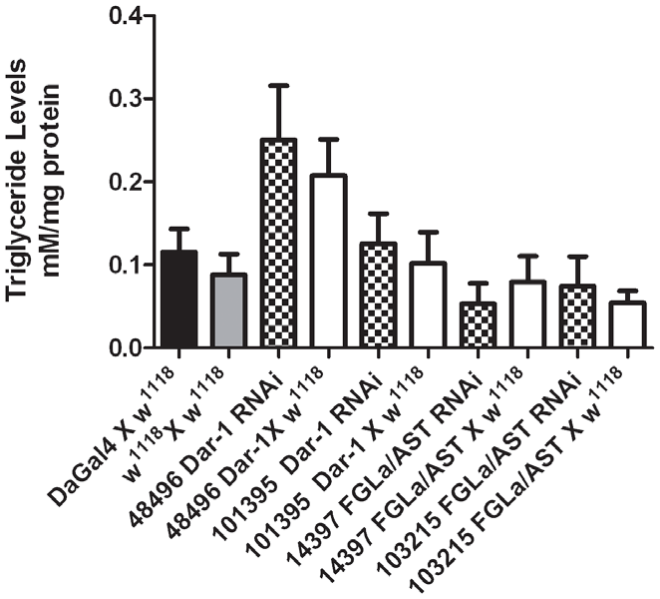
The triglyceride levels of third instar larvae in Dar-1 and FGLa/AST RNAi x DaGal4 and controls DaGal4 x *w^1118^* and *w^1118^* self-cross. Each bar represents the mean of three independent triglyceride assays. Triglycerides were extracted from five third instar larvae. Triglyceride levels were normalized using protein levels as a standard.

In contrast to our hypothesis, there was no significant difference in triglyceride levels between the Dar-1 and FGLa/AST RNAi lines crossed to DaGal4 or *w^1118^* (Figure 5).

## Discussion

FGLa/AST peptides were first identified as JH biosynthesis inhibitors from brain extracts of *Diploptera punctata* [8], [26]. In *D. punctata*, FGLa/ASTs have been shown to elicit myoinhibitory effects in the hindgut and activate midgut α-amylase secretion [12], [27]. Injection of FGLa/ASTs into *Blattella germanica* females inhibited food consumption, linking FGLa/ASTs with the regulation of digestive or feeding processes [28]. In *D. melanogaster*, FGLa/AST-specific antibodies reveal expression in interneurons, motorneurons and endocrine cells in the midgut [7]. FGLa/ASTs do not inhibit JH biosynthesis or innervate the ring gland/corpora allata in Diptera [3]. Based on immunohistochemical localization and mRNA expression, FGLa/ASTs are referred to as brain-gut peptides [29] but their function remains unclear. Work in *C. elegans* provided a testable hypothesis that *D. melanogaster* FGLa/ASTs and their CNS-localized receptor Dar-1 may be associated with foraging behavior [18]. Foraging behaviors in *D. melanogaster*, has been associated with naturally occurring variations in the *for* gene that encodes PKG [19], [30]. On food, the rover phenotype (*for^R^)* exhibits greater mobility than the sitter phenotype (*for^S^)*, however, both genotypes move at similar speeds in the absence of food [31], [32]. PKG has an evloutionary conserved function in the regulation of foraging behavior in fruit flies, the honeybee *Apis mellifera*, red harvester ant *Pogonomyrmex barbatus*, and the nematode*C. elegans* [33]–[35]. Nurse honey bees were found to have lower PKG activity levels and lower *Amfor* RNA levels (ortholog of *for*) than forager honey bees, as well, nurse honey bees can change to foragers when fed an activator of PKG [33]. Interestingly, the difference in PKG activity is reversed when comparing dwellers to roamers in *C. elegans*, where a loss-of-function mutation in PKG (*egl-4)* caused an increase in roaming behavior in the presence of food. This suggests that PKG has a conserved function among these organisms even though the effect it has may differ between them.

Our results show that the ubiquitous expression of FGLa/AST RNAi or Dar-1 RNAi is related to a decrease in foraging behavior of *D. melanogaster* third instar larvae in the presence of food. Foraging behavior, under these conditions, is not altered in the absence of food. This alteration in foraging behavior appears to be related to a decrease in *for* transcript levels of Dar-1 and FGLa/AST RNAi lines crossed to DaGal4 compared to both RNAi lines crossed to *w^1118^*. This suggests that *D. melanogaster* Dar-1 and its FGLa/AST ligand directly or indirectly activates or stabilizes *for* gene expression, as reduction of Dar-1 or its FGLa/AST ligand significantly reduces *for* mRNA levels causing a reduced foraging (*for^S^*) phenotype. RNAi lines crossed to *w^1118^* (i.e. no driver) appeared to have reduced *for* transcripts relative to controls DalGal4 X *w^1118^* and the *w^1118^*self-cross, however, this reduction was only significant in the case of 14397 FGLa/AST X *w^1118^* and may be explained, in part, by all RNAi lines X *w^1118^* having ‘leaky’ expression which led to a significant reduction in their respective gene expression. The decrease in foraging behavior on food was not found when the reduction in FGLa/AST and Dar-1 mRNA levels were reduced in a tissue selective manner. This is consistent with the observation that the tissue specific RNAi did not reduce Dar-1 or FGLa/AST gene expression to the levels caused by the ubiquitously expressed DaGal4 driver. Expression of Dar-1 and FGLa/AST RNAi under the direction of the tissue selective driver 6986 did not alter *for* mRNA levels relative to controls. It is also likely that foraging behavior is only affected when FGLa/ASTs interact with Dar-1 and alter *for* expression in select cellular localizations.

*C. elegans npr-9* mutants showed a significant increase in intestinal lipid accumulation compared to N2 wild type worms, which would suggest that the increased local search behavior seen in these mutant worms may also increase food uptake efficiency [18]. Since Dar-1 and FGLa/AST RNAi lines showed foraging defects similar to that of *npr-9* mutants, we hypothesized that similar increase in triglycerides would also be seen. However, our results show no significant difference in triglyceride levels between the Dar-1 and FGLa/AST RNAi lines crossed to either the DaGal4 driver or *w^1118^* control. The lack of lipid accumulation when Dar-1 or FGLa/AST levels are reduced is similar to lipid accumulation in *D. melanogaster for^S^* larvae. . In the presence of food, *for^R^* larvae ingest less food, exhibit higher glucose absorption and preferential glucose allocation to lipids rather than sugars. *for^R^* larvae thus have higher lipid levels than *for^S^* larvae [36], [37]. This contrasts with *A. mellifera*, where foraging bees have reduced lipid levels in comparison to nurse bees [38].

FGLa/ASTs and receptor Dar-1 do not participate in the regulation of juvenile hormone biosynthesis in *D. melanogaster* [39]. The alteration in foraging behavior and direct or indirect influence on the *for* transcript is the first function assigned to the *D. melanogaster* FGLa/ASTs and its receptor Dar-1. Future work will be directed at defining where FGLa/ASTs interact with Dar-1 and how this interaction influences *for* gene expression.
